# RAS GTPases are modified by SUMOylation

**DOI:** 10.18632/oncotarget.23269

**Published:** 2017-12-15

**Authors:** Byeong Hyeok Choi, Changyan Chen, Mark Philips, Wei Dai

**Affiliations:** ^1^ Department of Environmental Medicine, New York University Langone Medical Center, New York, NY, USA; ^2^ Department of Biochemistry and Molecular Pharmacology, New York University Langone Medical Center, New York, NY, USA; ^3^ Center for Drug Discovery, Northeastern University, Boston, MA, USA; ^4^ Department of Pathology, New York University Langone Medical Center, New York, NY, USA

**Keywords:** RAS, sumoylation, post-translational modification, oncogenesis

## Abstract

RAS proteins are GTPases that participate in multiple signal cascades, regulating crucial cellular processes including cell survival, proliferation, differentiation, and autophagy. Mutations or deregulated activities of RAS are frequently the driving force for oncogenic transformation and tumorigenesis. Given the important roles of the small ubiquitin-related modifier (SUMO) pathway in controlling the stability, activity, or subcellular localization of key cellular regulators, we investigated here whether RAS proteins are posttranslationally modified (*i.e.* SUMOylated) by the SUMO pathway. We observed that all three RAS protein isoforms (HRAS, KRAS, and NRAS) were modified by the SUMO3 protein. SUMOylation of KRAS protein, either endogenous or ectopically expressed, was observed in multiple cell lines. The SUMO3 modification of KRAS proteins could be removed by SUMO1/sentrin-specific peptidase 1 (SENP1) and SENP2, but not by SENP6, indicating that RAS SUMOylation is a reversible process. A conserved residue in RAS, Lys-42, was a site that mediates SUMOylation. Results from biochemical and molecular studies indicated that the SUMO-E3 ligase PIASγ specifically interacts with RAS and promotes its SUMOylation. Moreover, SUMOylation of RAS appeared to be associated with its activation. In summary, our study reveals a new posttranslational modification for RAS proteins. Since we found that HRAS, KRAS, and NRAS can all be SUMOylated, we propose that SUMOylation might represent a mechanism by which RAS activities are controlled.

## INTRODUCTION

RAS proteins are probably the mostly studied proto-oncogene products because of their frequent mutations in human malignancies. HRAS, KRAS and NRAS are three related gene products that are commonly expressed in mammalian cells and have overlapping but distinctive functions [1–4]. RAS proteins are GTPases that participate in multiple signal cascades, regulating crucial cellular processes including cell survival, proliferation, differentiation, and autophagy [2, 5, 6]. RAS proteins are membrane-anchored proteins whose activity, subcellular localization, and stability are tightly controlled as deregulated expression and/or its activities frequently lead to malignant transformation [1]. For example, deregulated activity of KRAS due to mutations plays a key role in the genesis of several types of common cancers [1–4].

RAS proteins are regulated by post-translational modifications include farnesylation, carboxylmethylation, and palmitoylation [1, 7–10]. Nascent RAS proteins are first processed by three-step modifications that lead to generation of lipidated proteins with hydrophobic C-termini that mediate association with cellular membranes [1]. RAS proteins are also modified by palmitoylation through covalent attachment of 16-carbon palmitoyl chain to cysteine residues. Palmitoylation is a necessary step for translocation of RAS proteins from the endomembrane system to the cell surface membrane [1, 7].

Extensive research in the past has also revealed that RAS proteins are subjected to other posttranslational modifications including phosphorylation, ubiquitination, acetylation, and S-nitrosylation [8, 11–15]. For example, KRAS4B is phosphorylated on serine 181 by protein kinase C and the phosphorylation is involved in the negative regulation of its association with the plasma membrane. RAS proteins are also modified by monoubiquitination and bi-ubiquitination [11–13]. Lys117, Lys147 and Lys170 are potential sites of ubiquitination [1]. The E3 ligase specific for RAS ubiquitination is RABEX5 [13]. Cys118, a highly conserved site in RAS isoforms and orthologues, can be modified by nitrosylation [16]. S-nitrosylation facilitates guanine nucleotide exchange, promoting efficient RAS activation [16, 17].

The SUMO (small ubiquitin-related modifier) pathway resembles the ubiquitin pathway. It consists of three enzymatic components including the E1 activating enzyme (SAE1/2), the E2 conjugating enzyme (UBC9) and a series of E3 ligases that promote SUMOylation in a substrate-specific manner [18, 19]. SUMOylation occurs on those lysine residues immediately after amino acids of a hydrophobic nature. Mammalian cells have three structural homologs: SUMO1, SUMO2, and SUMO3. Similar to other types of post-translational modifications, SUMOylation is reversed by the proteolytic cleavage of isopeptidases of the SENP family [19]. Extensive research has uncovered important functions of SUMOylation that control the subcellular localization, stability and enzyme activities of target proteins. SUMOylation is also critical in development and cell biology, as its disruption either causes abnormal cellular growth and differentiation or leads to embryonic lethality [20].

Given that SUMOylation plays a major role in regulating stability, activity, and subcellular localization, as well as in modulating RAS signaling pathway [21], we investigated whether RAS proteins were post-translationally modified by SUMOs. Using a combination of biochemical and molecular approaches, we observed that HRAS, KRAS and NRAS proteins were all SUMO-modified and that SUMO3 were the most-efficient modifier. Moreover, Lysine 42 (K42) was an important residue for regulating SUMOylation and PIASγ is an E3 ligase promoting RAS sumoylation *in vitro*. Significantly, sumoylation appears to be important for RAS activation.

## RESULTS

### Ectopically expressed RAS proteins are modified by SUMOylation

**Figure 1:**
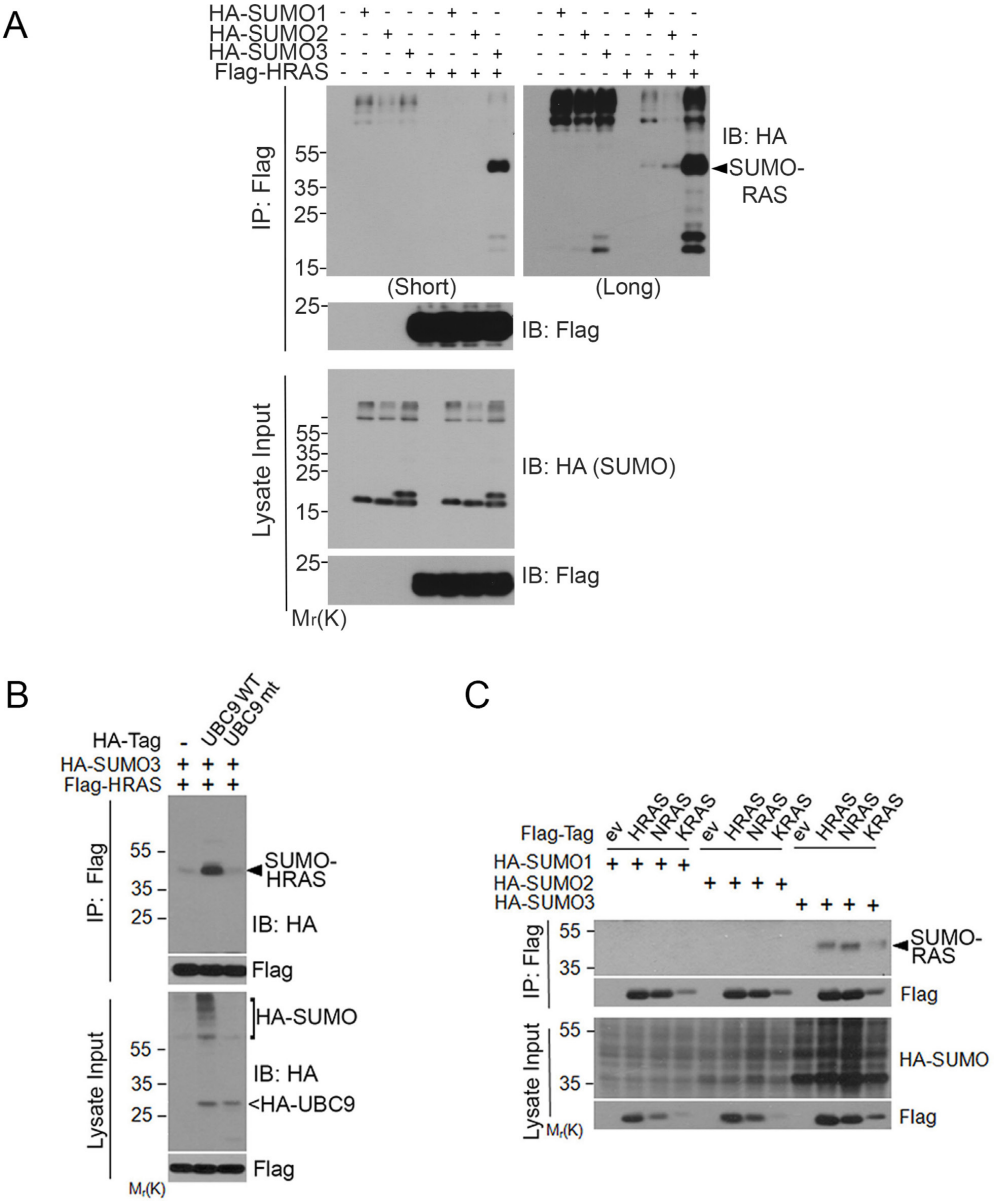
RAS proteins are post-translationally modified by SUMO3 (**A**) HEK293 cells were co-transfected with Flag-HRAS and HA-SUMO isoforms as indicated. Equal amounts of protein lysates from various treatments were immunoprecipitated with the anti-Flag antibody. Flag immunoprecipitates, along with lysate inputs, were blotted with either anti-HA antibody or anti-Flag antibody. (**B**) HEK293 cells were co-transfected with expression constructs of Flag-HRAS, HA-SUMO3, and UBC9 (WT) or UBC9 inactive mutant (mt). Equal amounts of protein lysates from various treatments were immunoprecipitated with the anti-Flag antibody. Flag immunoprecipitates, along with lysate inputs, were blotted with the anti-HA antibody or the anti-Flag antibody. (**C**) HEK293T cells were co-transfected with constructs expressing Flag-RAS isoforms (HRAS, NRAS, and KRAS4B) and HA-SUMO isoforms (SUMO1, SUMO2, and SUMO3) as indicated. Equal amounts of protein lysates from various treatments were immunoprecipitated with the anti-Flag antibody. Flag immunoprecipitates, along with lysate inputs, were blotted with the anti-Flag antibody or with the anti-HA antibody.

Given that SUMOylation plays a major role in regulating stability, activity, and subcellular localization and that UBC9, the SUMO E2 conjugating enzyme in mammalian cells [22], is required for oncogenesis driven by the RAS/Raf pathway [23], we investigated whether RAS proteins were post-translationally modified by SUMOs. We first ectopically expressed Flag-HRAS and HA-tagged SUMO1, SUMO2, or SUMO3. We observed that co-expression of Flag-HRAS and HA-SUMO3 resulted in a slow-mobility band that migrated between 35 and 55 kDa (Figure 1A). The molecular weight was about the size of HRAS conjugated with one SUMO3 moiety. Longer exposure revealed that there was a small amount of modified signal at the same location when HA-SUMO1 or HA-SUMO2 was co-expressed with Flag-HRAS. Three isoforms of SUMO and transfected HRAS (lysate inputs) were expressed at comparable levels among different transfection samples, suggesting that HRAS might be primarily modified by HA-SUMO3.

To confirm that HRAS was modified by SUMO3, we co-transfected cells with Flag-HRAS and HA-SUMO3, along with the wild-type (WT) or catalytically inactive mutant of UBC9, the E2 enzyme required for SUMOylation in mammalian cells. We found that Flag immunoprecipitates contained significant amounts of SUMO3-modified HRAS when cells were transfected with WT UBC9, but not mutant UBC9 (Figure 1B). HA-UBC9 expression was confirmed by blotting with the anti-HA antibody.

To determine whether SUMO-modification was unique to HRAS, we transfected cells with Flag-KRAS4B (named KRAS thereafter), Flag-NRAS or Flag-HRAS expression constructs, along with HA-tagged SUMO1, SUMO2 or SUMO3. We found that immunoprecipitates of all three isoforms of RAS contained SUMO3, but not SUMO1- or SUMO2 (Figure 1C), strongly suggesting that RAS proteins are primarily modified by SUMO3 in an isoform-dependent manner. Although the level of SUMOylated KRAS was relatively low, this was consistent with the lower level of overall Flag-KRAS expression.

### Endogenous RAS proteins are SUMO-modified

**Figure 2:**
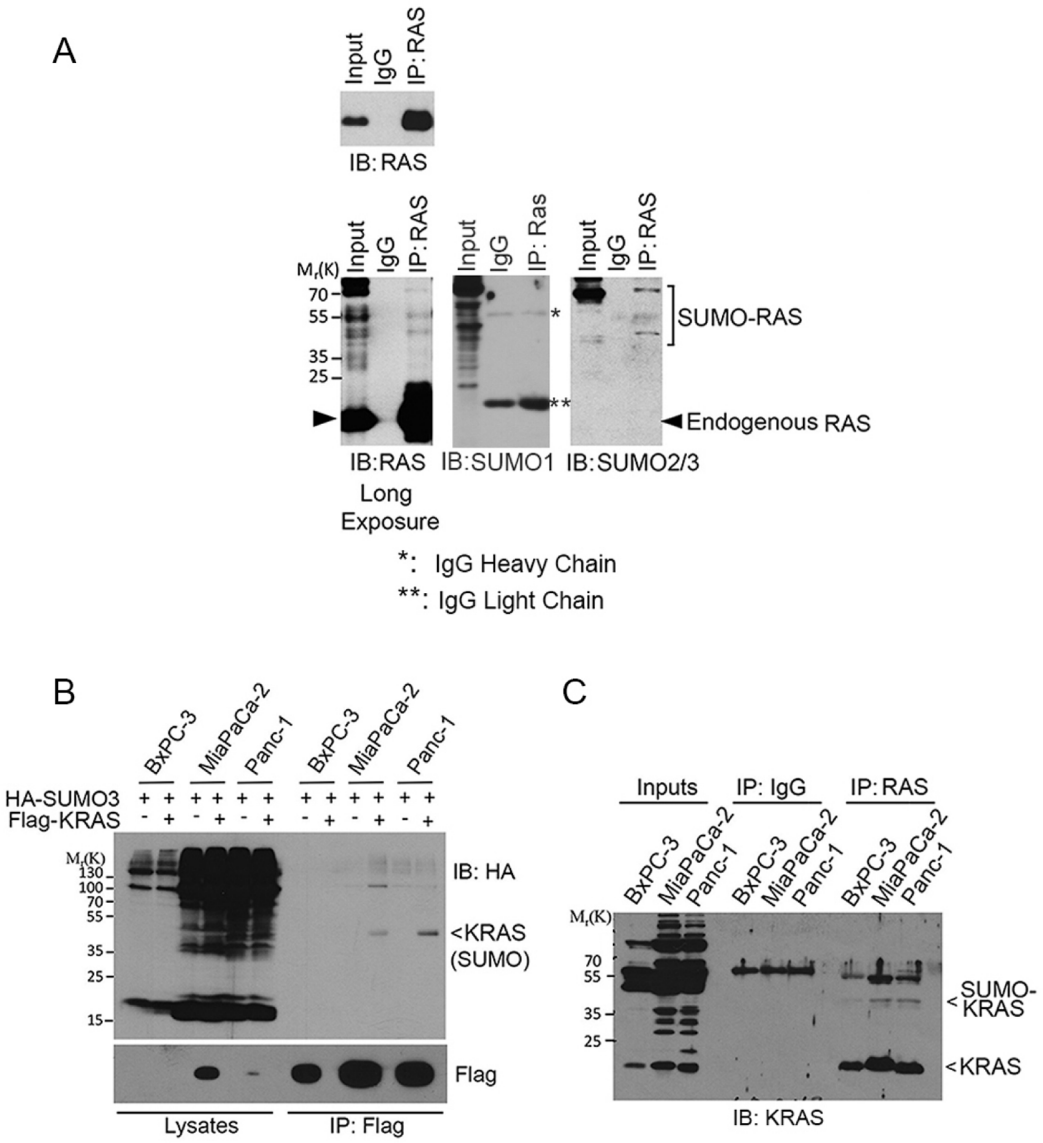
SUMOylation of endogenous RAS proteins (**A**) HEK293T cell lysates were incubated with bead immobilized with mouse monoclonal anti-RAS IgGs or with control IgGs. Proteins bound to the bead, along the lysate input, were eluted and blotted with a rabbit polyclonal anti-RAS antibody or with anti-SUMO1 and anti-SUMO2/3 antibodies. Both short (upper panel) and long (lower panel) exposures of RAS blot were shown. Endogenous native RAS and SUMOylated RAS are indicated. IgG light (^**^) and heavy (^*^) chains are also indicated. (**B**) Pancreatic cell lines including BxPC-3 (wild-type KRAS), MiaPaCa-2 and Panc-1 (KRAS^V12^) were transfected with Flag-KRAS and HA-SUMO3 for 24 h, after which equal amounts of cell lysates were precipitated with the anti-Flag antibody. Flag immunoprecipitates, along with cell lysates, were then blotted with the HA antibody. (**C**) BxPC-3, MiaPaCa-2 and Panc-1 cells were lysed and equal amounts of cell lysates were incubated with bead immobilized with mouse monoclonal anti-RAS IgGs or with control IgGs. Proteins bound to the bead, along the lysate inputs, were blotted with a rabbit polyclonal anti-KRAS4B antibody. Both endogenous native KRAS and SUMOylated KRAS are indicated.

To determine whether endogenous RAS was also SUMOylated, we immunoprecipitated HEK293T cell lysates with an anti-RAS antibody or with control IgG. Blotting immunoprecipitates with both anti-RAS, anti-SUMO1, and anti-SUMO2/3 antibodies revealed that slow-mobility RAS-specific bands were detectable that co-migrated with SUMO-modified signals only when the immunoprecipitates were blotted with the SUMO2/3 antibody but not the SUMO1 antibody. These observations strongly suggest that these bands are SUMO2/3-modified RAS (Figure 2A).

KRAS plays a crucial role in mediating oncogenesis in pancreas [24]. We next determined whether KRAS SUMOylation occurred in pancreatic cell lines. BxPC-3, MiaPaCa-2, and Panc-1 cells were transfected with plasmids expressing Flag-KRAS and HA-SUMO3 for 24 h. Flag-immunoprecipitates were blotted with HA. We observed that SUMOylation of ectopically expressed KRAS was easily detectable in MiaPaCa-2 and Panc-1 cells (Figure 2B). Subsequent studies revealed that endogenous KRAS was SUMOylated in all three pancreatic cell lines although SUMO-modified KRAS signals were weaker in BxPC-3 cells than those in MiaPaCa-2 and Panc-1 (Figure 2C). Of note, BxPC-3, but not MiaPaCa-2 and Panc-1, cells contain WT KRAS [25, 26].

**Figure 3:**
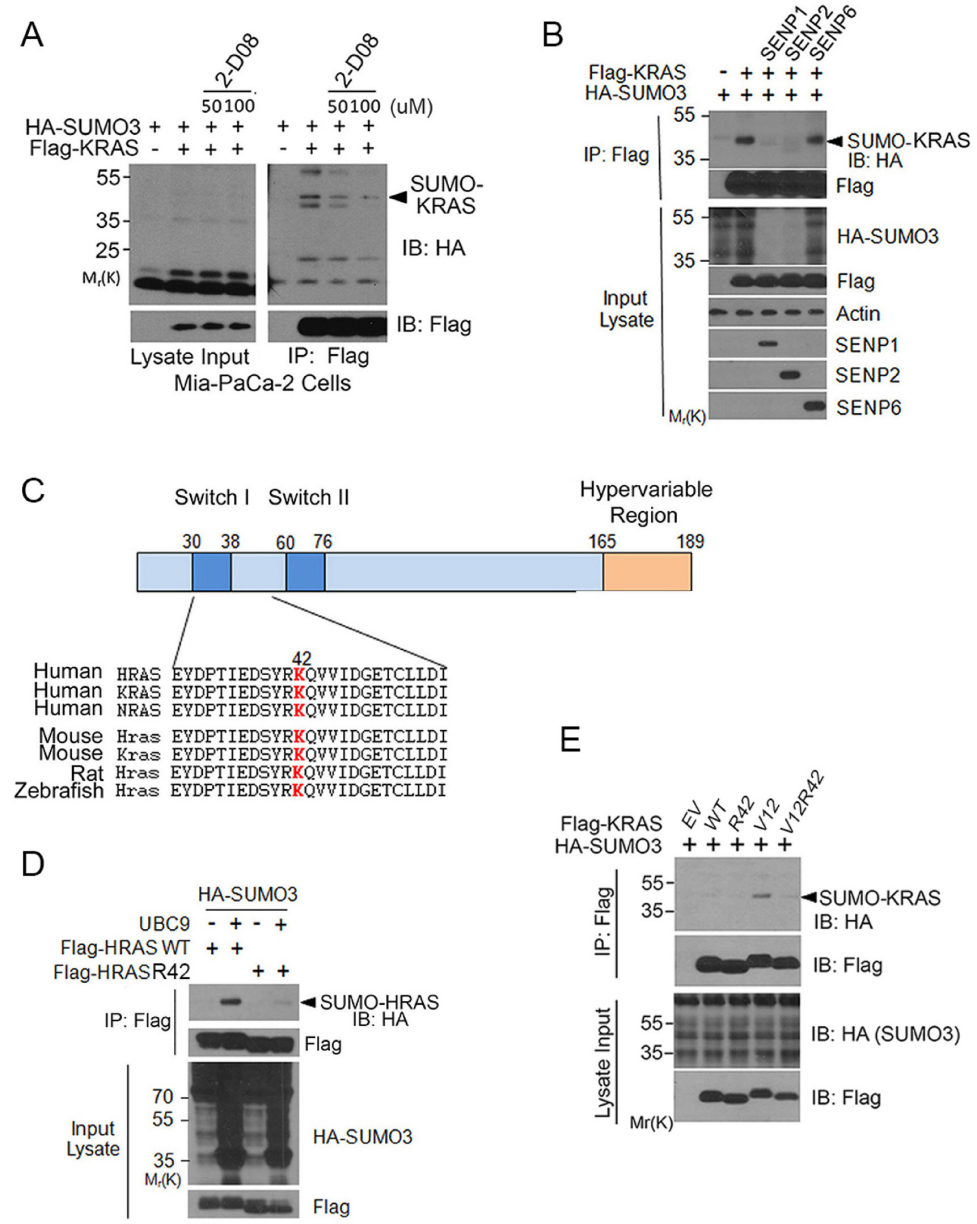
K42 is a key residue regulating RAS SUMOylation (**A**) MiaPaCa-2 cells were transfected with Flag-KRAS and HA-SUMO3 expression constructs for 24 h and then treated with or without 2-D08 for 18 h. Cells were then lysed and equal amounts of the lysates were immunoprecipitated with the anti-Flag antibody. Flag precipitates, along with lysate inputs, were blotted with antibodies to HA and Flag, respectively. (**B**) HEK293T cells were co-transfected with plasmid constructs expressing Flag-KRAS, HA-SUMO3, and SENP isoforms as indicated. Equal amounts of protein lysates were immunoprecipitated with the anti-Flag antibody. Flag immunoprecipitates were immunoblotted with antibodies to Flag or HA. Protein lysates of various treatments were also blotted with antibodies to HA, Flag, β-actin, SENP1, SENP2, or SENP6. (**C**) Alignment of RAS isoform amino acid sequences with a predicted SUMOylation site (in red). The residue for optimal sumoylation was predicted with SUMOplot (http://www.abgent.com/sumoplot). (**D**) HEK293T cells were co-transfected with constructs expressing Flag-tagged HRAS (WT) or HRAS^42R^, HA-UBC9, and HA-SUMO3. Equal amounts of protein lysates from various treatments were immunoprecipitated with the anti-Flag antibody. Flag immunoprecipitates, along with lysate inputs, were blotted with the anti-Flag antibody or with the anti-HA antibody. (**E**) HEK293T cells were co-transfected with HA-SUMO3 and WT KRAS or with HA-SUMO3 and various forms of KRAS mutants as indicated for 24 h. Equal amounts of cell lysates were immunoprecipitated with the anti-Flag antibody. Flag immunoprecipitates, along with cell lysate inputs, were blotted with antibodies to HA and Flag, respectively.

To further validate that KRAS was SUMOylated, we treated cells transfected with Flag-KRAS expression plasmid with 2-D08, a SUMO E2 inhibitor [27]. We noted that 2-D08 blocked KRAS SUMOylation in a concentration-dependent manner (Figure 3A). To determine whether KRAS SUMOylation was a reversible process, cells were co-transfected with plasmid constructs expressing Flag-KRAS, HA-SUMO3, and an isopeptidase (SENP1, SENP2, or SENP6) that was capable of removing a specific SUMO moiety from its substrates [28]. We observed that expression of SENP1 or SENP2, but not SENP6, abolished SUMOylated KRAS (Figure 3B), indicating that SENP1 and SENP2 are likely isopeptidases that desumoylate KRAS *in vivo*.

### Lysine 42 is a major site for regulating RAS SUMOylation

To identify a potential lysine residue(s) that was responsible for, or involved in regulating, SUMOylation, we scanned RAS protein sequences optimal for SUMOylation using an on-line software (SUMOplot). We noted that K42 was one of several sites potential for mediating SUMOylation and that this residue was highly conserved (Figure 3C). We first substituted arginine (R) for lysine (K) at this position and found a marked reduction in incorporation of HA-SUMO3 (Figure 3D), consistent with the notion that K42 may be an important site of SUMOylation or mediating SUMOylation. Because K104 is also a site potential for SUMOylation and this site is also known for RAS acetylation [14], we asked whether K42 SUMOylation was affected by the status of K104. K104 mutation alone somewhat compromised HRAS SUMOylation; however, additional K42 mutation did not further reduce residual signals of SUMOylation (Supplementary Figure 1).

To confirm that K42 plays a role in mediating RAS SUMOylation, we co-expressed HA-SUMO3 and Flag-KRAS, Flag-KRAS^V12^, or Flag-KRAS^V12/R42^ in HEK293T cells. Flag immunoprecipitates were blotted with the anti-HA antibody. We observed that KRAS^V12^ contained an easily detectable amount of SUMO-modified signals and that mutation at K42 significantly reduced KRAS SUMOylation (Figure 3E). Expression of KRAS and various mutants was comparable.

**Figure 4:**
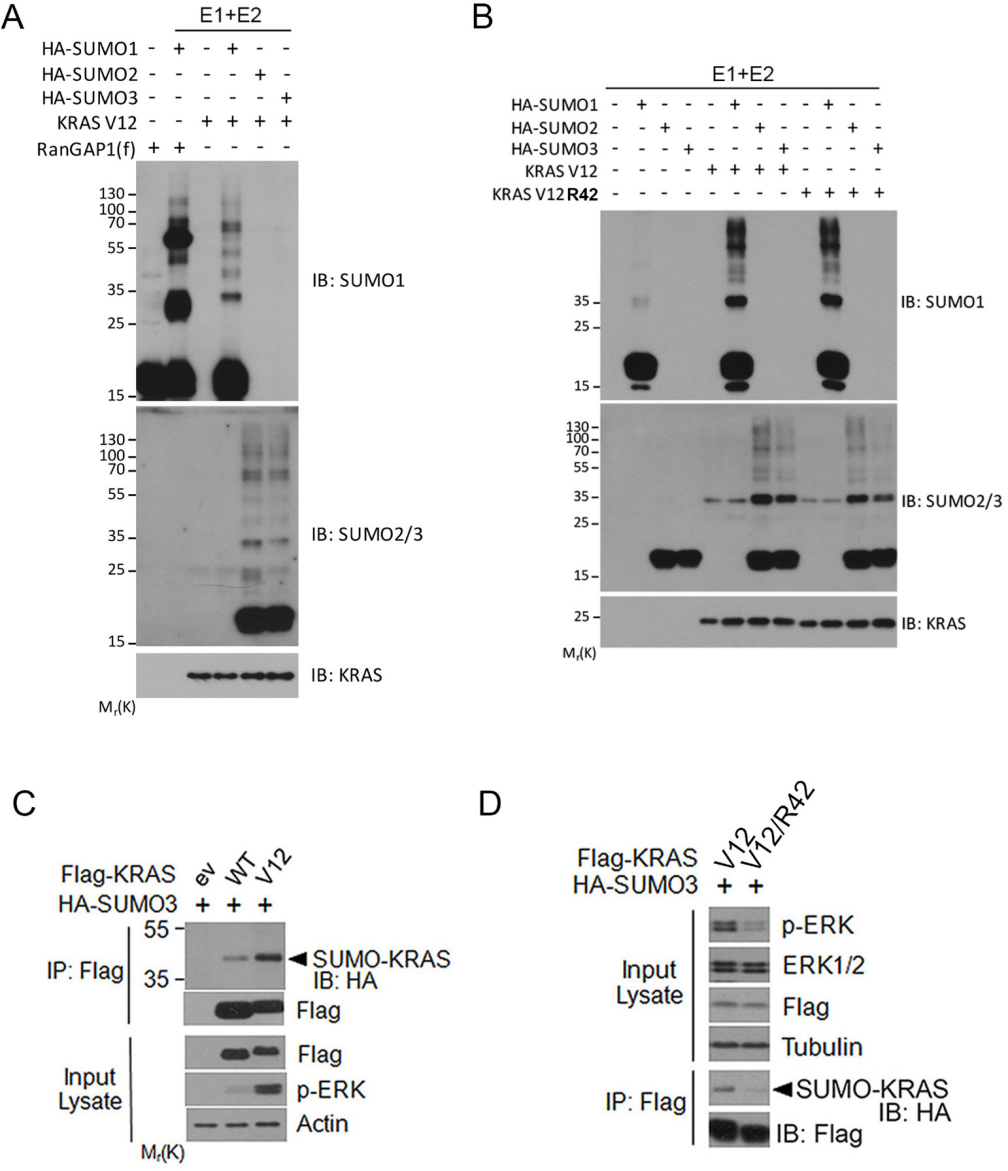
Analysis of KRAS SUMOylation *in vitro* (**A**) *In vitro* SUMOylation assays were performed with defined components including E1, E2, SUMO isoforms, and substrate KRAS^V12^ as described in Experimental Procedures. RanGAP1 was used as positive control substrate. *In vitro* reactions were blotted with antibodies to SUMO1, SUMO2, and RAS protein, respectively. (**B**) *In vitro* SUMOylation assay carried out using KRAS^V12^ or KRAS^V12/R42^ as substrates. At the end of reaction, samples were blotted with antibodies to SUMO1, SUMO2/3, and RAS protein, respectively. (**C**) HEK293T cells were co-transfected plasmid constructs expressing Flag-KRAS (either WT or V12) and HA-SUMO3 for 24 h after which cells were collected and lysed. Equal amounts of cell lysates were precipitated with the anti-Flag antibody. Flag immunoprecipitates were blotted with the anti-Flag or anti-HA antibody. Lysates were also blotted with antibodies to Flag, phospho-ERK (p-ERK), and β-actin. (**D**) HEK293T cells were co-transfected plasmid constructs expressing Flag-KRAS^V12^ (or KRAS^V12/R42^) and HA-SUMO3 for 24 h, after which cell lysates were immunoprecipitated with the anti-Flag antibody. Flag immunoprecipitates were blotted with the anti-Flag antibody or the anti-HA antibody. Lysates were also blotted with antibodies to p-ERK, total ERK, Flag, and α-tubulin.

To further confirm that RAS proteins are modified by SUMOylation, we carried out *in vitro* reconstitution assays using purified proteins. We observed that addition of all three isoforms of SUMO to the assays led to their incorporation into KRAS protein (Figure 4A), indicating that KRAS can be modified by all three isoforms of SUMO *in vitro*. In addition to mono-sumoylated band, a few other high molecular weight bands were also detected, suggesting KRAS proteins can be modified at multiple residues *in vitro* as well. Our subsequent assays revealed that whereas KRAS modification *in vitro* by HA-SUMO2 and HA-SUMO3, but not HA-SUMO1, was significantly affected by K42 mutation in KRAS protein (Figure 4B).

### RAS SUMOylation regulates its activity

To study whether RAS SUMOylation affected its activity through modulating downstream signaling, we first analyzed ERK activation in cells expressing transfected KRAS (either wild-type or V12 mutant). As expected, expression of mutant KRAS^V12^ resulted in higher levels of SUMOylation than that of WT KRAS (Figure 4C). Significantly, compared with KRAS^V12^, expression of SUMO-resistant mutant KRAS^V12/42R^ greatly reduced p-ERK signals despite that their expression was similar (Figure 4D). These results strongly suggest that RAS SUMOylation is associated with its activation.

### PIASγ plays an major role in mediating RAS SUMOylation

**Figure 5:**
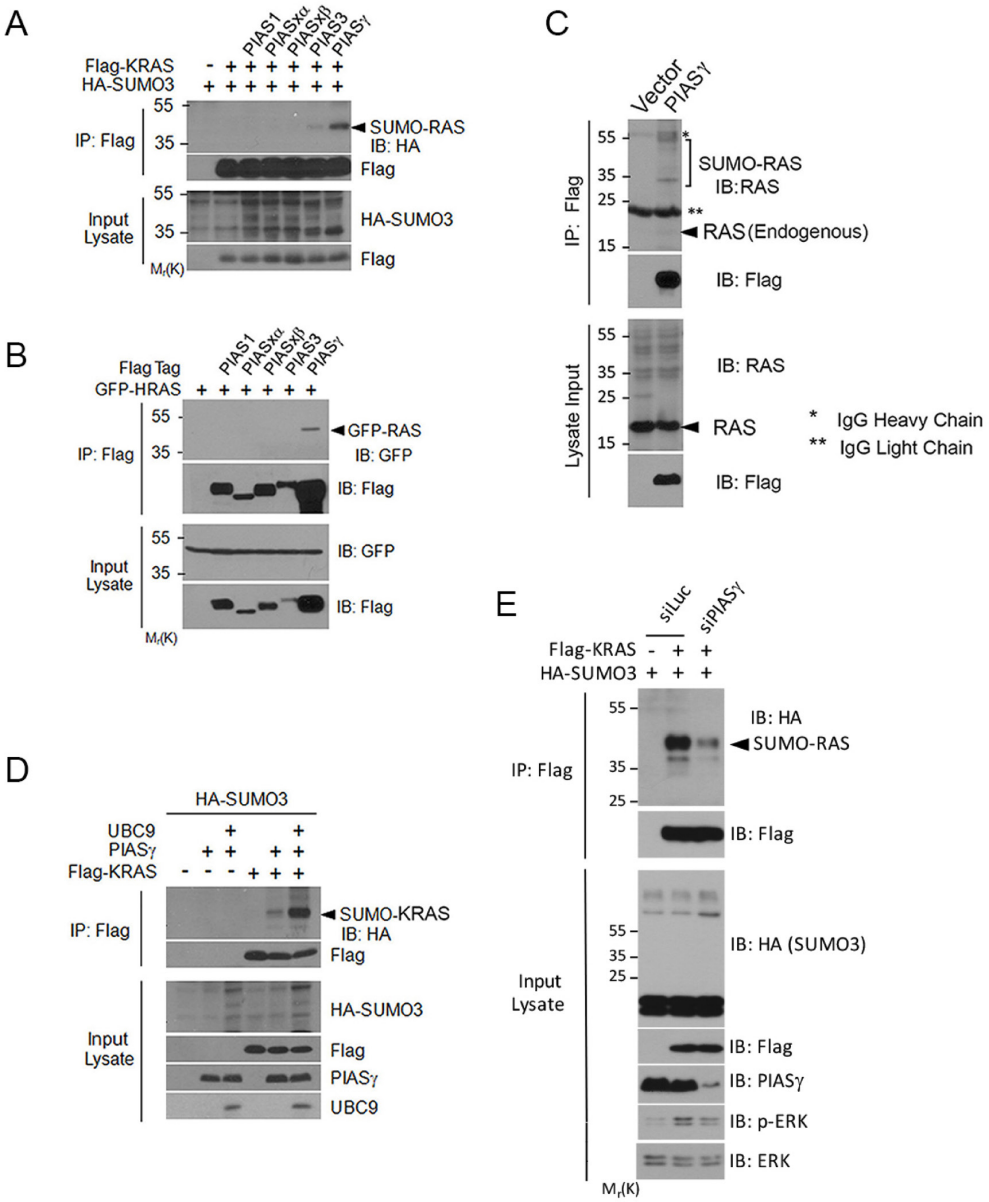
PIASγ is SUMO E3 ligase for RAS proteins (**A**) HEK293T cells were co-transfected with plasmid constructs expressing Flag-tagged proteins of the PIAS family, Flag-KRAS and/or HA-SUMO3. Equal amounts of protein lysates from various treatments were immunoprecipitated with the anti-Flag antibody. Flag immunoprecipitates, along with the lysate inputs, were immunoblotted with the anti-Flag or the anti-HA antibody. (**B**) HEK293T cells were co-transfected with individual Flag-tagged PIAS expression constructs and GFP-HRAS expression construct. Equal amounts of protein lysates were immunoprecipitated with the anti-Flag antibody. Flag immunoprecipitates, along with lysate inputs, were immunoblotted with an anti-GFP antibody or anti-Flag antibody. (**C**) HEK293T cells were co-transfected with plasmid constructs expressing Flag-PIASγ or vector) and UBC9. Equal amounts of protein lysates were immunoprecipitated with the anti-Flag antibody. Flag immunoprecipitates, along with lysate inputs, were blotted with antibodies to Flag and RAS. Endogenous RAS and IgGs (light and heavy chains) are indicated. (**D**) HEK293T cells were transfected with plasmid constructs expressing HA-SUMO3, Flag-KRAS, PIASγ, and/or UBC9 for 24 h after which cells were collected and lysed. Equal amounts of cell lysates were immunoprecipitated with the Flag antibody. Immunoprecipitates, along with lysate inputs, were blotted with antibodies to HA, Flag, PIASγ, and/or UBC9. (**E**) HEK293T cells were co-transfected with Flag-KRAS and/or HA-SUMO3 expression constructs, and siRNAs to either PIASγ (siPIAS) or luciferase (siLuc) for 24 h as indicated, after which cells were collected and lysed. Equal amounts of cell lysates were immunoprecipitated with the Flag antibody. Flag immunoprecipitates were blotted with antibodies to HA and Flag, respectively. Corresponding cell lysates were also blotted with antibodies to HA, Flag, PIASγ, total ERKs, and p-ERKs, respectively.

To identify a potential SUMO E3 ligase(s) for RAS, we ectopically expressed various genes of the PIAS family [29, 30] and determined which gene product(s) was capable of stimulating KRAS SUMOylation. We observed that expression of PIASγ significantly stimulated KRAS SUMOylation although PIAS3 also induced a low level of SUMOylation (Figure 5A), suggesting that PIASγ may be a likely SUMO E3 for KRAS. Consistent with this observation, PIASγ (PIAS4) is required for conjugating SUMO2/3 to protein substrates during DNA damage responses [31]. Expression of KRAS and various PIAS family members was comparable as revealed by blotting with the anti-Flag antibody. PIASγ precipitates, but not pull-down materials of other members of the PIAS family, contained significant amounts of HRAS signals (Figure 5B), suggesting the physical interaction between HRAS and PIASγ. Moreover, ectopically expressed PIASγ was capable of pulling-down endogenous RAS protein (Figure 5C).

In addition, expression of PIASγ enhanced KRAS SUMOylation, which was further stimulated by expression of UBC9 (Figure 5D). Significantly, downregulation of PIASγ, caused compromised activation of KRAS activity, leading to weakened phosphorylation of ERKs (Figure 5E, p-ERK). These results strongly suggest that PIASγ is a *bona fide* SUMO E3 ligase for RAS proteins and that SUMOylation positively regulates its activity.

## DISCUSSION

RAS proteins are among the most potent oncogenic gene products, which directly impact upon the MAP kinase signaling pathway. In this study, we report for the first time that RAS proteins can be modified by SUMOylation and that SUMOylation appears to be associated with its activation. We have observed that there are several salient features of RAS SUMOylation: (1) All three isoforms of RAS proteins (HRAS, KRAS and NRAS) are SUMOylated; (2) whereas all three SUMO isoforms (SUMO1, SUMO2, and SUMO3) can be conjugated to RAS proteins *in vitro* SUMO3 is primary component that is conjugated to RAS proteins *in vivo*; (3) Lysine 42 (K42) plays a major role in mediating RAS SUMOylation; (4) RAS SUMOylation is a general phenomenon, occurring in all cell types that are examined; (5) SUMOylation is involved in regulating RAS activity, affecting downstream signaling.

K42 is a highly conserved residue among all three RAS isoforms. As HRAS, KRAS and NRAS can be all modified by SUMO, we speculate that SUMOylation may represent a fundamental mechanism by which RAS activities and/or their subcellular localization is controlled. Similar to di-ubiquitination, modification with SUMO3 is likely to affect subcellular localization of RAS proteins. An earlier study showed that *Drosophila* RAS1 was modified by SUMO1 at K104; however, mutation of K104 into R did not drastically reduce RAS1 sumoylation [32], suggesting that additional lysine residues may be major accepting sites for SUMO moiety. Future studies will be focused on the mode of action of SUMOylation in regulating subcellular localizations (e.g., cell surface and intracellular membranes) of RAS proteins, as well as physical interaction between RAS proteins and downstream components. It is also noted that K42 lies in close proximity to RAS Switch I domain, which is important for mediating its interaction with Raf. It is possible that K42 mutation may cause a conformation change, which in turn, affect the interaction between RAS and SUMO-conjugating enzyme(s), thus compromising its SUMOylation.

HRAS is activated by monoubiquitination and ubiquitination accelerates intrinsic nucleotide exchange, thereby promoting GTP loading [12, 33]. Despite that our study reveals no major difference between wild-type and SUMO-resistant mutant, we speculate that SUMOylation may facilitate the plasma membrane and/or endosome association of RAS, leading to its activation. It has been shown that among the isoforms of RAS, there are differences in the mode of actions in their activation by monoubiquitination [33, 34]. HRAS activity is promoted by monoubiquitination at K117 through accelerating intrinsic nucleotide exchange, leading to enhanced GTP loading; on the other hand, KRAS monoubiquitination at K147 results in compromised GTP hydrolysis [33, 34]. The net effect of monoubiquitination on these two lysine sites, therefore, remains the same with more GTP-bound, active RAS protein.

There are significant differences of SUMO isoform incorporation into RAS proteins between *in vivo* and *in vitro* analyses. *In vitro* SUMOylation assays show that all three isoforms of SUMO can be incorporated into RAS proteins at comparable levels. However, only SUMO3 is efficiently conjugated to RAS proteins *in vivo*. It is possible that accessary factors within the cell may mediate fine-tuning of RAS modifications by SUMO isoforms.

KRAS is the major driver of human cancers. It is generally agreed that KRAS^V12^ mutant is constitutively locked in the active, GTP-bound state, which makes it very challenging to inhibit its oncogenic activity through traditional drug design approaches. It appears feasible to disrupt the association between RAS and its downstream effectors via a small molecular compound, thus blocking its signaling and activity [35]. Given that KRAS is SUMO-modified *in vivo* and that SUMO inhibitor (2-D08) is capable of blocking its SUMOylation, our current studies may provide a new avenue of research into possible inactivation of KRAS oncogenic activity through the use of small molecular chemical compounds.

## MATERIALS AND METHODS

### Cell culture and transfection

HEK293T and pancreas adenocarcinoma (BxPC3, MiaPaCa-2 and Panc-1) cell lines were obtained from the American Type Culture Collection and they were cultured in DMEM supplemented with 10% fetal bovine serum (FBS, Invitrogen) and antibiotics (100 μg/ml of penicillin and 50 μg/ml of streptomycin sulfate, Invitrogen) at 37°C under 5% CO_2_. Transfection was achieved with either LF2000 (Invitrogen) or Fugene HD (Roche Diagnostics) following the manufacturers’ protocols. Transfection efficiency was estimated to be between 80–100% in all cases through transfecting a GFP expressing plasmid (Data not shown).

### Plasmids and reagents

HA-tagged SUMO1, SUMO2, SUMO3, wild-type UBC9, UBC9 mutant, Flag-tagged PIAS1, PIASxa, PIASxß, PIAS3, PIASγ, SENP1, SENP2 and SENP6 were obtained from Addgene. Flag-PIAS3 was kind gifts from Dr. Angeliki Malliri. Individual RAS mutants were obtained using a Quikchange Site-directed mutagenesis kit (Agilent Technologies). All mutations were confirmed by DNA sequencing. SUMOylation inhibitor (2-D08) was purchased from Sigma (SML1052).

### Protein expression and purification

The BL21(DE3) strains of *E. coli* were transformed with the plasmid(s) of interest, and successful transformants were selected on LB agar [1.5% (w/v)] with Ampicillin (50 μg/mL) for pET-14b KRAS^V12^ and pET-14b KRAS^V12/K42^. The culture was incubated at 37°C with agitation until the OD600 reached 0.5, at which point IPTG was added to 0.4 mM to induce expression of target genes. The cultures were then incubated at 16°C for 16 h.

At the end of incubation, bacteria were collected by centrifugation (30 min at 20,000 × g at 4°C). Bacterial pellets were frozen at −80°C for 1 h, and then thawed to ensure complete lysis. The pellets were resuspended in 5 mL lysis buffer [10 mM TrisHCl (pH 8.0), 100 mM NaH_2_PO4 (pH 8.0), 10 mM imidazole, 150 mM NaCl, 0.25% (v/v) Triton X-100, protease inhibitor cocktail (P8340, Sigma), 1 mM dithiothreitol (DTT), 10mg/ml Lysozyme (L6876, Sigma) and 3 units/ml Benzonase (E1014, Sigma)]. The cell resuspension was incubated on ice for 30 min with occasional vortexing. The resuspension was then lysed by sonication in 35% output, 30 sec bursts and 30 sec resting on ice between each burst for 5 min. The insoluble fraction was removed by centrifuging at 20000 × g for 30 min at 4°C.

Lysates were used to purify recombinant proteins by affinity chromatography using Ni-NTA agarose (QIAGEN^®^ 30210, which is a 50% slurry). After removing the insoluble fraction from the lysate, Ni-NTA agarose was washed 3 times with lysis buffer and added 1:50 ratio (v/v), and incubated for 16 h, rocking at 4°C. The resin was pelleted by centrifugation (500 g, 30 sec), then washed 3 times with washing buffer [10 mM TrisHCl (pH 8.0), 100 mM NaH_2_PO4 (pH 8.0), 150 mM NaCl, 20 mM imidazole]. The eluate fraction (Purified protein) was collected from the supernatant after incubating the resin for 15 min with an elution buffer [10 mM TrisHCl (pH 8.0), 100 mM NaH_2_PO4 (pH 8.0), 150 mM NaCl, 500 mM imidazole]. Eluted samples were loaded onto a PD-10 desalting column (17–0851, GE Healthcare) with the final buffer of 20 mM TrisHCl (pH 8.0) and 150 mM NaCl. Purified proteins were used for *in vitro* assays after quantification.

### *In vitro* SUMOylation assay

*In vitro* SUMOylation assay was carried out at 37°C for 1 h using a SUMOylation kit (Enzo Life Science) and purified His-KRAS^V12^ or KRAS^K42^ proteins. For each reaction, His-KRAS or the mutant proteins (250 ng) were incubated with the following proteins: SUMO E1 (25 ng), SUMO E2 (25 ng), SUMO1 (500 ng), SUMO2 (500 ng), and/or SUMO3 (500 ng).

### Immunoprecipitation and immunoblotting

Cells were lysed in TBSN buffer [20 mM Tris-Cl (pH 8.0), 150 mM NaCl, 0.5% NP-40, 5 mM EGTA, 1.5 mM EDTA, 0.5 mM Na_3_VO_4_, and 20 mM β-Glycerol phosphate]. Cell lysates were clarified by centrifugation at 15,000 × g for 20 min at 4°C. Cleared lysates were incubated with anti-Ras antibody (Abcam ab16907) or control IgG for overnight at 4°C. Protein A/G plus-agarose beads (Santa Cruz) were added to each sample. After 1 h, the beads were washed three times with lysis buffer, followed by immunoblotting. For Flag- immunoprecipitation, Cells were lysed in TBSN buffer [20 mM Tris-Cl (pH 8.0), 150 mM NaCl, 0.5% NP-40, 5 mM EGTA, 1.5 mM EDTA, 0.5 mM Na_3_VO_4_, and 20 mM β-glycerol phosphate]. The cell lysates were clarified by centrifugation at 15,000 × g for 20 min at 4°C. Cleared lysates (1 mg) were added to Flag M2 agarose (Sigma) followed by incubation in the TBSN buffer for 1 h at 4°C. After incubation, proteins bound to each resin were washed extensively with the lysis buffer, eluted in the SDS-PAGE sample buffer, and analyzed by SDS-PAGE. Total cell lysates were prepared in a buffer [50 mM Tris-HCl (pH 7.5), 150 mM NaCl, 1% IGEPAL, 0.1% SDS, and 0.5% sodium deoxycholate] supplemented with a mixture of protease and phosphatase inhibitors. Protein concentrations were measured using the bicinchoninic acid protein assay reagent kit (Pierce Chemical Co). Equal amounts of protein lysates from various samples were used for SDS–PAGE analysis followed by immunoblotting. Antibodies specific for phospho-ERK42/44 (Cell Signaling, 4370), ERK1/2 (Cell Signaling, 4695), HA (Cell Signaling, 3724), Flag (Cell Signaling, 2368), Ras (Abcam, ab52939), SUMO1 (Enzo life science, BML-PW9460), SUMO2/3 (Enzo life science, BML-PW9465), KRAS (Santa Cruz, Sc-521), Actin (Cell Signaling, 4970), Ras for mouse monoclonal (Abcam ab16907) and PIASγ (Cell Signaling, 4392) were purchased from the indicated companies. Antibody to GFP, SENP1, SENP2 and SENP6 were purchased from Santa Cruz Biotechnology. Specific signals on immunoblots (polyvinylidene difluoride) were visualized using enhanced chemiluminescence (Pierce Chemical Co.).

## SUPPLEMENTARY MATERIALS FIGURE


