# Supplementary material for: RAS GTPases are modified by SUMOylation
Source: Oncotarget. 2017 Dec 15;9(4):4440–50. doi: 10.18632/oncotarget.23269 (PMC5796985; doi:10.18632/oncotarget.23269)
Supplement: Supplementary file 1 [file oncotarget-09-4440-s001.pdf]

RAS GTPases are modified by SUMOylation

SUPPLEMENTARY MATERIALS

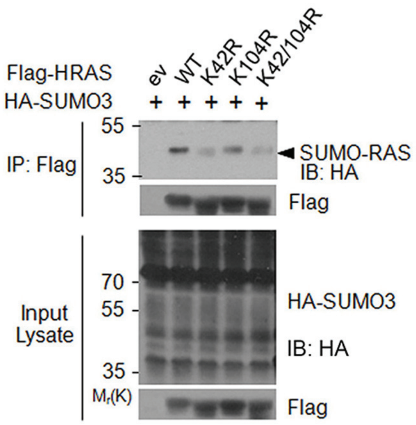

Supplementary Figure 1: HEK293T cells were co-transfected with plasmid constructs expressing Flag-HRAS (WT) or various mutants as indicated (HRAS^{42R} HRAS^{104R} or HRA$^{42R/104R}$) and HA-SUMO3 for 24 h. Equal amounts of protein lysates from various transfection were immunoprecipitated with the anti-Flag antibody. Flag immunoprecipitates, along with lysate inputs, were blotted with the anti- Flag antibody or with the anti-HA antibody.
